# Danshensu Attenuates Palmitic Acid-Induced Activation of Hepatic Stellate Cells by Regulating Pyroptosis

**DOI:** 10.7150/ijms.107564

**Published:** 2025-03-19

**Authors:** Han-Fang Tseng, Huan-Nung Chao, Chih-Hung Lin, Chan-Yen Kuo

**Affiliations:** 1 Department of Anesthesiology, Taichung Veterans General Hospital, Taichung 407, Taiwan.; 2 Department of Nephrology, Hanming Christian Hospital, Changhua City 500, Taiwan.; 3 Department of Internal Medicine, Cathay General Hospital, Taipei 106, Taiwan.; 4 School of Medicine, College of Medicine, Fu Jen Catholic University, New Taipei City 242, Taiwan.; 5 Department of Research, Taipei Tzu Chi Hospital, Buddhist Tzu Chi Medical Foundation, New Taipei City 231, Taiwan.; 6 Department of Nursing, Cardinal Tien College of Healthcare and Management, New Taipei City 231, Taiwan.; 7 Institute of Oral Medicine and Materials, College of Medicine, Tzu Chi University, Hualien 970, Taiwan.

**Keywords:** connective tissue growth factor, hepatic stellate cells, inflammasomes, palmitic acid, pyroptosis, *Salvia miltiorrhiza*

## Abstract

**Introduction:** We focused on examining the role of Danshensu in reducing reactive oxygen species (ROS) production and inhibiting NLRP3 inflammasome activation, which are key factors in liver fibrosis and inflammation. We sought to explore the potential of Danshensu as a therapeutic agent for liver fibrosis by targeting the pyroptosis-inflammasome signaling pathway, providing a basis for developing effective and safer NLRP3 inflammasome inhibitors. This study aimed to investigate whether Danshensu can mitigate palmitic acid (PA)-induced activation of hepatic stellate cells (HSCs) by regulating pyroptosis in HSC-T6 and LX-2 cells.

**Methods:** HSC-T6 and LX-2 cell lines served as the cell models. A 2',7'-dichlorofluorescin diacetate reagent was used to measure ROS production within cells. Cell protein extraction was performed using radioimmunoprecipitation assay lysis buffer. The protein concentration in each sample was measured using a BCA assay kit. Western blot analysis was used with the SDS-polyacrylamide gel electrophoresis system.

**Results:** PA-induced activation of HSC-T6 and LX-2 cells by upregulating alpha-smooth muscle actin, integrin-β1, and connective tissue growth factor. Danshensu mitigated PA-induced ROS accumulation in these cells. Moreover, Danshensu potentially reversed the upregulation of NLRP3, cleaved caspase 1, interleukin-1, GSDME, and ASC in PA-activated LX-2 cells via pyroptosis, suggesting its therapeutic potential. Pyroptosis inhibitor tetramethylthiuram disulfide reversed Danshensu attenuated PA activation of HSC-T6 and LX-2 cells, resulting in a 2-fold increase in alpha-smooth muscle actin, integrin-β1, and connective tissue growth factor.

**Conclusion:** Danshensu effectively attenuates PA-induced HSC activation by reducing ROS production and inhibiting pyroptosis, offering a potential therapeutic strategy for liver fibrosis.

## Introduction

Chronic liver fibrosis should be studied extensively because it causes progressive damage to the liver and deaths worldwide 1. As liver fibrosis progresses, chronic liver injury activates hepatic stellate cells (HSCs) 2. Fibrosis of the liver occurs when extracellular matrix proteins are abnormally accumulated by the activated HSCs of the liver 3. In an activated, myofibroblast-like state, HSCs exhibit proliferation, contractility, fibrogenicity, chemotaxis, and immunomodulation 4,5. HSC activation concepts currently in development focus on novel mediators, intracellular signals, and factors that promote inactivation. Together, these concepts provide a framework for discovering new therapeutic targets 5.

Despite the reasonable prospects for treating liver fibrosis with Traditional Chinese Medicine herbs, their complex composition and wide range of targets make studying their antihepatic fibrosis mechanisms challenging 6. Danshen (*Salvia miltiorrhiza*) is commonly used in Traditional Chinese Medicine for treating cardiovascular diseases, cancers, liver diseases, and nervous system disorders 7. The active compounds found in Danshen and its extracts are prominently protective, anti-inflammatory, antioxidative, or antiplatelet in nature 8.

The inflammatory process of pyroptosis is caused by microbial infection, which activates inflammasomes and releases pro-inflammatory cytokines, such as interleukin-1β and interleukin-18 9,10. During pyroptosis, plasma membrane pores form as a result of caspase 1-dependent activation, which results in pathological ion fluxes that ultimately cause cellular lysis and inflammation 11. Stellate cells are activated, and liver fibrosis is induced by hepatocyte pyroptosis and inflammasome particle release 12. The TLR4-NF-κB signaling pathway is activated in HSCs by palmitic acid (PA) and activates the NLRP3 inflammasome. The activation of NLRP3 inflammasomes in liver stellate cells aggravates nonalcoholic steatohepatitis-induced liver fibrosis 13. It is therefore possible to treat liver fibrosis by targeting the pyroptosis-inflammasome signaling pathway. While pyroptosis, a form of programmed cell death, has been implicated in liver diseases and fibrosis, its relationship with activated HSCs is poorly understood.

This study aimed to investigate whether Danshensu can mitigate PA-induced activation of HSCs by regulating pyroptosis in T6 and LX-2 cells. The focus was on examining the role of Danshensu in reducing reactive oxygen species (ROS) production and inhibiting the activation of the NLRP3 inflammasome, which are key factors in liver fibrosis and inflammation. We aimed to explore the potential of Danshensu as a therapeutic agent for liver fibrosis by targeting the pyroptosis-inflammasome signaling pathway, thereby providing a basis for developing effective and safer NLRP3 inflammasome inhibitors.

## Methods

### Reagents

Palmitic Acid (PA; Sigma-Aldrich, St. Louis, MO, USA), Danshensu (Sigma-Aldrich, St. Louis, MO, USA), Tetramethylthiuram disulfide (TMTD; Disulfiram, Sigma-Aldrich, St. Louis, MO, USA) was used.

### Cell culture

The HSC-T6 cell line, derived from rat HSCs and acquired from Millipore (Billerica, MA, USA), served as the primary cell model in this investigation. Cultured under 5% CO_2_ in a humidified environment at 37°C, HSC-T6 cells were nurtured in Dulbecco's Minimum Essential Medium (Gibco, New York, NY, USA) supplemented with 10% fetal bovine serum and antibiotics (1,100 U/mL penicillin, 100 μg/mL streptomycin, and 2.5 μg/mL amphotericin B). The culture medium underwent replacement every 48 hours to maintain optimal cell conditions. Upon reaching 70-80% confluency, cells were detached using trypsinization and subsequently seeded into either 6-well or 24-well plastic dishes for downstream experiments. Passages 3-10 of the cell cultures were ultimately utilized in the study.

The LX-2 human HSC line was purchased from Sigma-Aldrich (Burlington, MA, USA). The cell line was grown in Dulbecco's Modified Eagle Medium (Gibco) containing 2% fetal bovine serum (Gibco) and 1% penicillin/streptomycin (Gibco) and incubated at 37°C with 5% CO_2_.

### Cells treatment with PA, Danshensu, and TMTD

Cells were pretreated with 0, 100, 200, and 400 μM PA for 24 h. And then, cells were treated with 30 μM Danshensu in presence or absence 2 μg/mL TMTD for another 24 h.

### Intracellular ROS generation measurement

Cells were washed with phosphate-buffered saline (PBS; Sigma) and incubated with 10 μM 2',7'-dichlorofluorescin diacetate (DCF-DA; Sigma) at 37 °C for 30 minutes in the dark. Upon exposure to reactive oxygen species (ROS), DCF-DA undergoes oxidation, resulting in fluorescence. Following incubation, the cells were trypsinized and rinsed three times with ice-cold PBS. Finally, ROS level was determined using flow cytometry (FACScan; Becton Dickinson, Franklin Lakes, NJ, USA) using an excitation wavelength of 498 nm and an emission wavelength of 522 nm.

### Protein extraction and western blot analysis

Cell protein extraction was performed using radioimmunoprecipitation assay lysis buffer containing protease inhibitor cocktail (Roche, Burlington, MA, USA) and phosphatase inhibitor cocktail (Roche), with a reaction time of 30-60 min on ice. The protein concentration in each sample was measured using a BCA assay kit (Thermo Fisher Scientific, Rockford, IL, USA).

Western blot analysis was used with the SDS-polyacrylamide gel electrophoresis system and afterwards transferred onto PVDF membranes of 0.45-µm pore size. Then, membranes were blocked with 5% milk or Bovine Serum Albumin (Sigma-Aldrich) in 1× TBST buffer for 30-60 min. Primary antibodies including rabbit polyclonal antibodies against NLRP3, caspase-1, GSDME, interleukin-1, ASC, GAPDH (1:1,000 dilution; ABclonal, Woburn, MA, USA), and β-actin (1:3,000 dilution; Cell Signaling, Danvers, MA, USA) were incubated with the membranes overnight at 4°C for probing. On alternate days, membranes were washed with 1× TBST buffer for 10 min, repeating the process 3 times. They were then incubated with a secondary antibody conjugated to horseradish peroxidase for 60 min at 25 °C. The membranes were washed with 1× TBST buffer for 10 min, repeating the process 3 times. All cell protein levels were analyzed by Cytiva Amersham™ ECL™ Prime Western Blotting Detection Reagent (Thermo Fisher Scientific) and a ChemiDocTMXRS+ System (Bio-Rad Laboratories, Hercules, CA, USA). The intensities of the protein bands were analyzed using Image J software.

### Statistical analysis

Statistical analyses of all data were performed using GraphPad Prism 5.0 (GraphPad Software, Boston, MA, USA), and a *P*-value of <0.05 was statistically significant. The multiple groups were analyzed by a one-way ANOVA test followed by a Tukey post-hoc test. Data were expressed as the mean ± standard error of the mean. Statistical significance was defined as a *P*-value of <0.05 in all tests.

## Results

### PA-activated HSC activation was reversed by Danshensu

**Figure 1:**
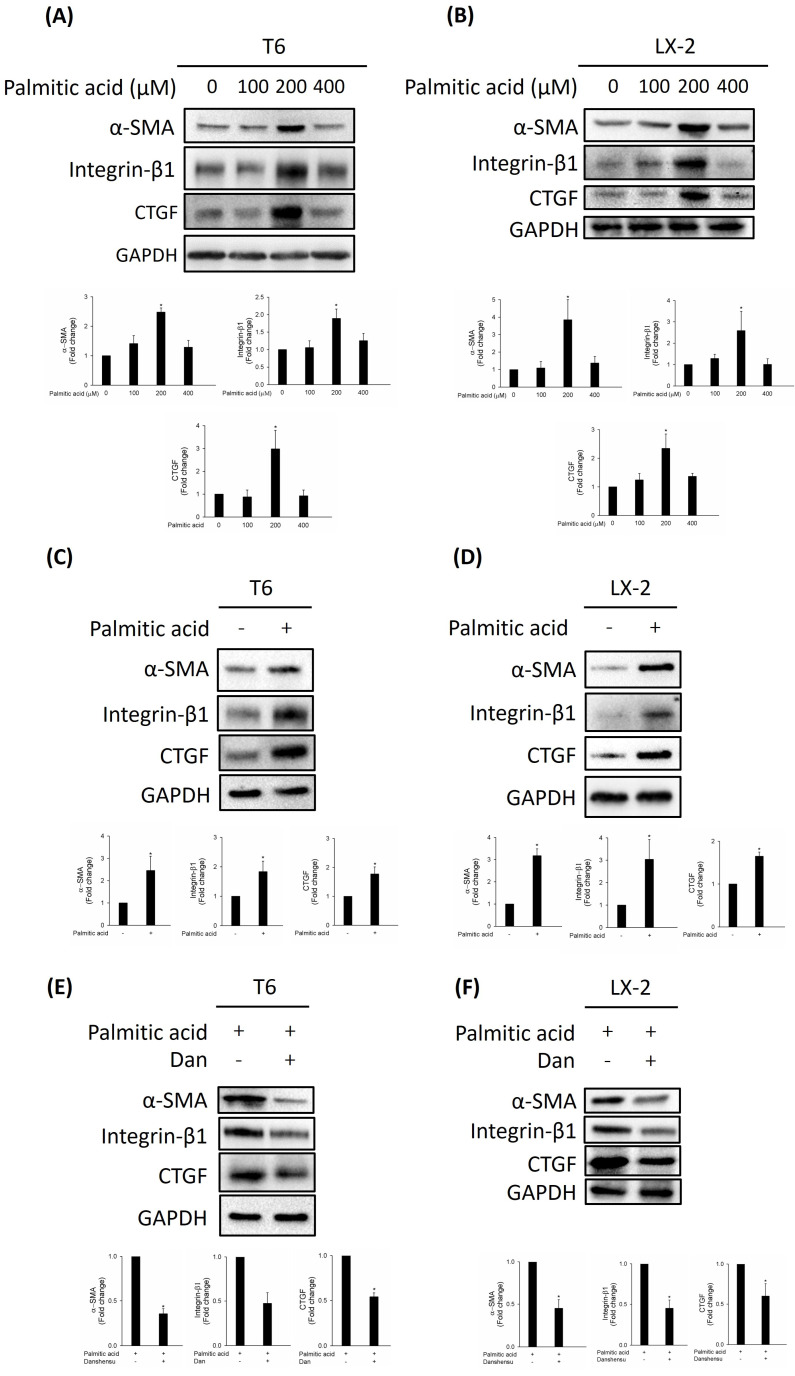
** Effect of Danshensu on palmitic acid (PA)-induced hepatic stellate cell activation via detection of alpha-smooth muscle actin (α-SMA), integrin-β1, and connective tissue growth factor (CTGF) expressions.** Change in the protein levels of α-SMA, integrin-β1, and CTGF after various concentrations of PA (0, 100, 200, and 400 μM) treatment in (A) T6 and (B) LX-2 cells in the absence of 30 μM Danshensu treatment. Change in the protein levels of α-SMA, integrin-β1, and CTGF after PA (200 μM) treatment in (C) T6 and (D) LX-2 cells in the presence of 30 μM Danshensu treatment. Lower panels indicated the quantitative results for specific proteins, which were determined using ImageJ. The data are presented as the mean ± SD from three independent experiments, with significance levels indicated as follows: *P < 0.05.

HSCs are activated by PA through inflammasome activation and hedgehog signaling 14. Increased alpha-smooth muscle actin (α-SMA), integrin-β1, and connective tissue growth factor (CTGF) expressions are the most reliable indicators of HSC activation 15. To study the effect of PA on HSC activation, we detected the expressions of activated HSC markers α-SMA, integrin-β1, and CTGF in various concentrations of PA (0, 100, 200, and 400 μM). Our results indicated that PA (200 μM) dramatically increased the expressions of α-SMA and CTGF in T6 and LX-2 cells (Fig. 1A, B). To further confirm the HSCs were activated by PA at 200 μM, we detected the expressions of α-SMA, integrin-β1, and CTGF in T6 and LX-2 cells. As shown in Figure 1C, D, upregulated α-SMA, integrin-β1, and CTGF were detected in T6 and LX-2 cells after 200 μM PA treatment for 24 h. We evaluated whether Danshensu has an anti-fibrotic effect on PA-activated T6 and LX-2 cells. Results indicated that an increase in the expressions of α-SMA, integrin-β1, and CTGF in PA-treated T6 and LX-2 cells was attenuated by Danshensu treatment (Fig. 1E, F). Taken together, PA triggered the activation of HSCs, a process that was subsequently counteracted by Danshensu.

### Danshensu alleviates ROS overproduction in PA-activated HSCs

**Figure 2:**
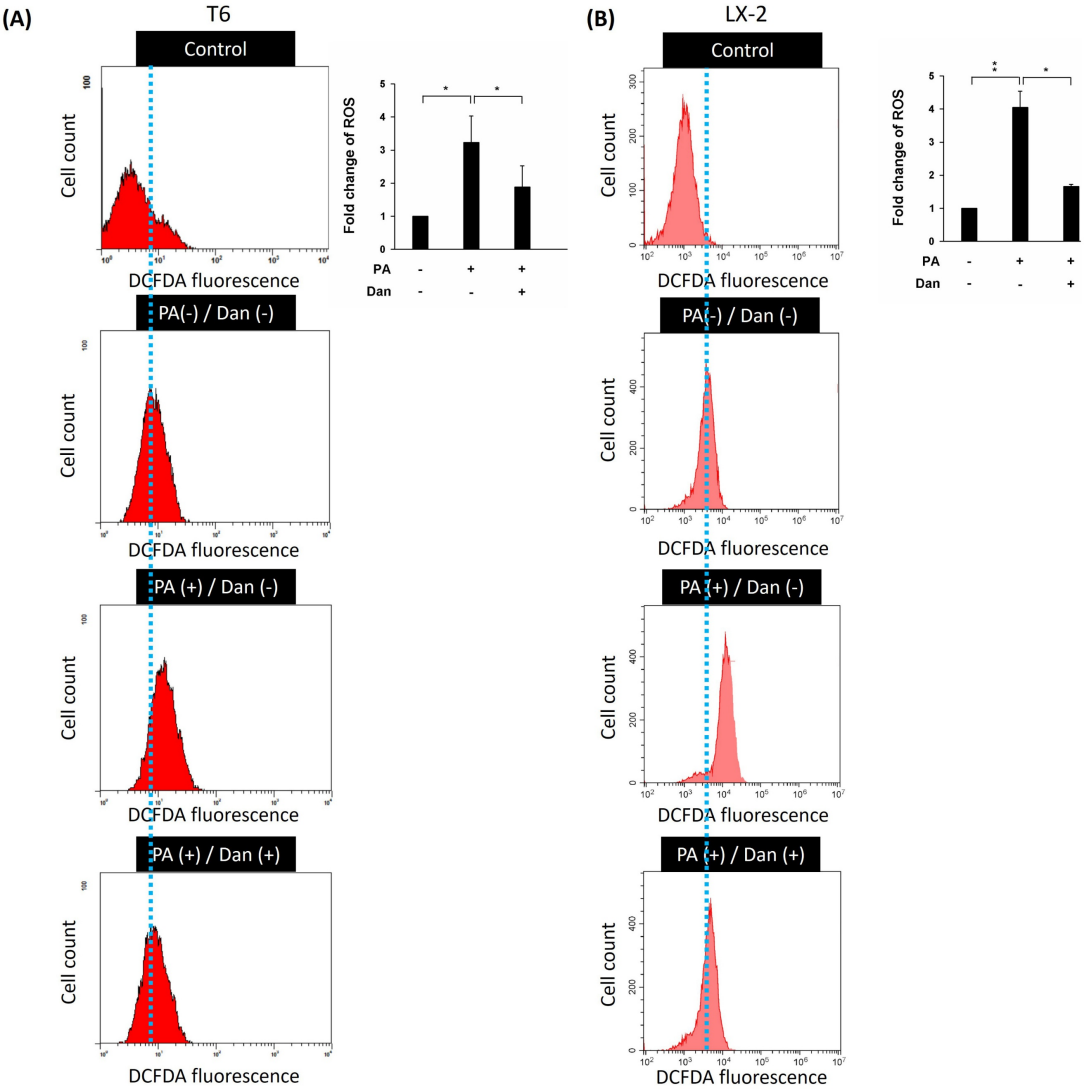
** Effect of Danshensu on reactive oxygen species (ROS) levels in palmitic acid (PA)-activated hepatic stellate cells.** Changes in ROS levels with 10 μM DCFDA staining or without DCFDA treatment (control), and in the absence (PA-) or presence (PA+) of 200 μM PA treatments with 30 μM (Dan+) or without (Dan-) Danshensu treatment in (A) T6 and (B) LX-2 cells. Right panels indicated the quantitative results for specific proteins, which were determined using ImageJ. The data are presented as the mean ± SD from three independent experiments, with significance levels indicated as follows: *P < 0.05.

**Figure 3:**
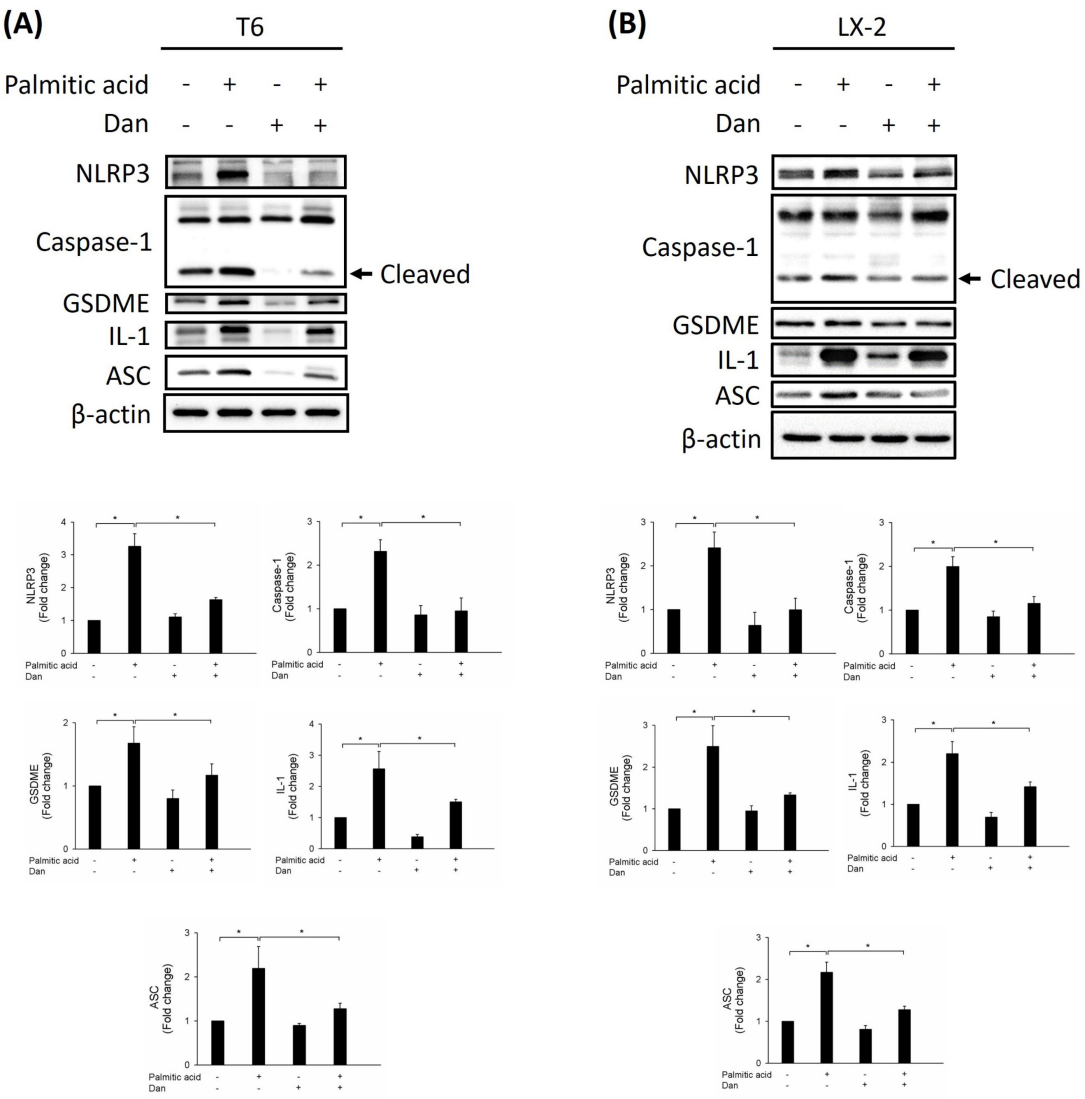
** Effect of Danshensu on pyroptosis in palmitic acid (PA)-activated hepatic stellate cells.** Changes in the expression of NLRP3, caspase-1, GSDME, interleukin-1, and ASC in the absence (PA-) or presence (PA+) of 200 μM PA treatments with 30 μM (Dan+) or without (Dan-) Danshensu treatment in (A) T6 and (B) LX-2 cells. Lower panels indicated the quantitative results for specific proteins, which were determined using ImageJ. The data are presented as the mean ± SD from three independent experiments, with significance levels indicated as follows: *P < 0.05.

Due to their ability to produce ROS, mitochondria are potent immune system stimulators and interact with the NLRP3 inflammasome during inflammation. ROS damage mitochondrial DNA and produce inflammation 16,17. ROS generated by mitochondria with reduced membrane potential can activate the NLRP3 inflammasome. Rotenone-blocking complex I prevented ROS generation and inflammasome activation 18. Additionally, the study demonstrated that the NLRP3 inflammasome plays a key role in the progression of liver fibrosis 19. Therefore, we hypothesize that Danshensu alleviated PA-activated LX-2 and T6 cells by decreasing the levels of ROS and inactivation of the NLRP3 inflammasome. Results indicated that the levels of ROS were increased in PA-treated T6 and LX-2 cells. Conversely, Danshensu attenuated PA-induced ROS overproduction (Fig. 2) and NLRP3 upregulation (Fig. 3) in T6 and LX-2 cells. Overall, Danshensu reduced ROS production and decreased NLRP3 expression in PA-activated HSCs.

### Danshensu attenuates PA-activated HSCs via pyroptosis

**Figure 4:**
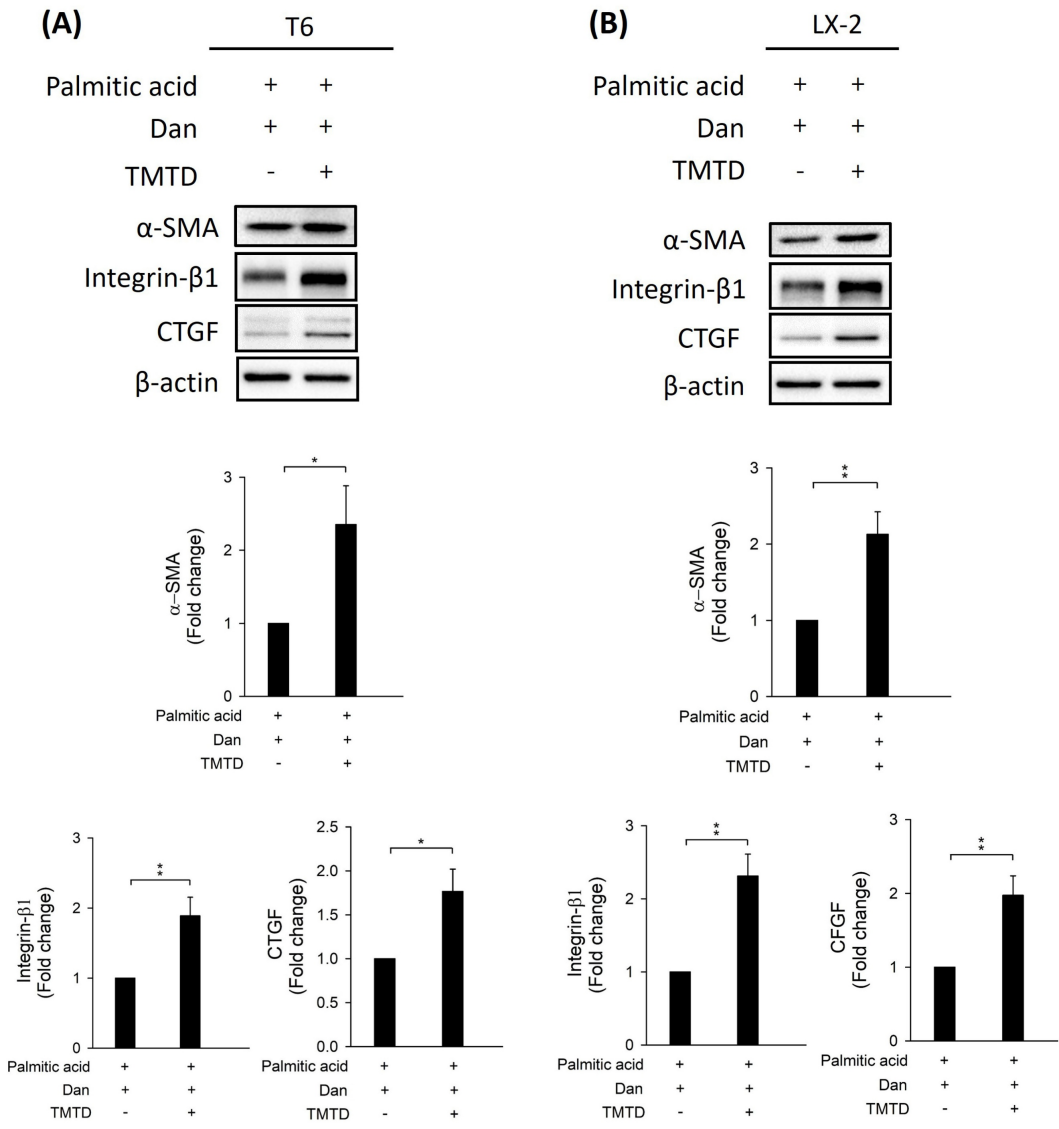
** Effect of tetramethylthiuram disulfide (TMTD) on Danshensu attenuated palmitic acid (PA)-hepatic stellate cell activation via detection of alpha-smooth muscle actin (α-SMA), integrin-β1, and connective tissue growth factor (CTGF) expressions.** Upper panels indicated change in the protein levels of α-SMA, integrin-β1, and CTGF after PA (200 μM) and 30 μM Danshensu treatment in (A) T6 and (B) LX-2 cells with or without 2 μg/mL TMTD treatment. Lower panels indicated the quantitative results for specific proteins, which were determined using ImageJ. The data are presented as the mean ± SD from three independent experiments, with significance levels indicated as follows: *P < 0.05, **P < 0.01.

**Figure 5:**
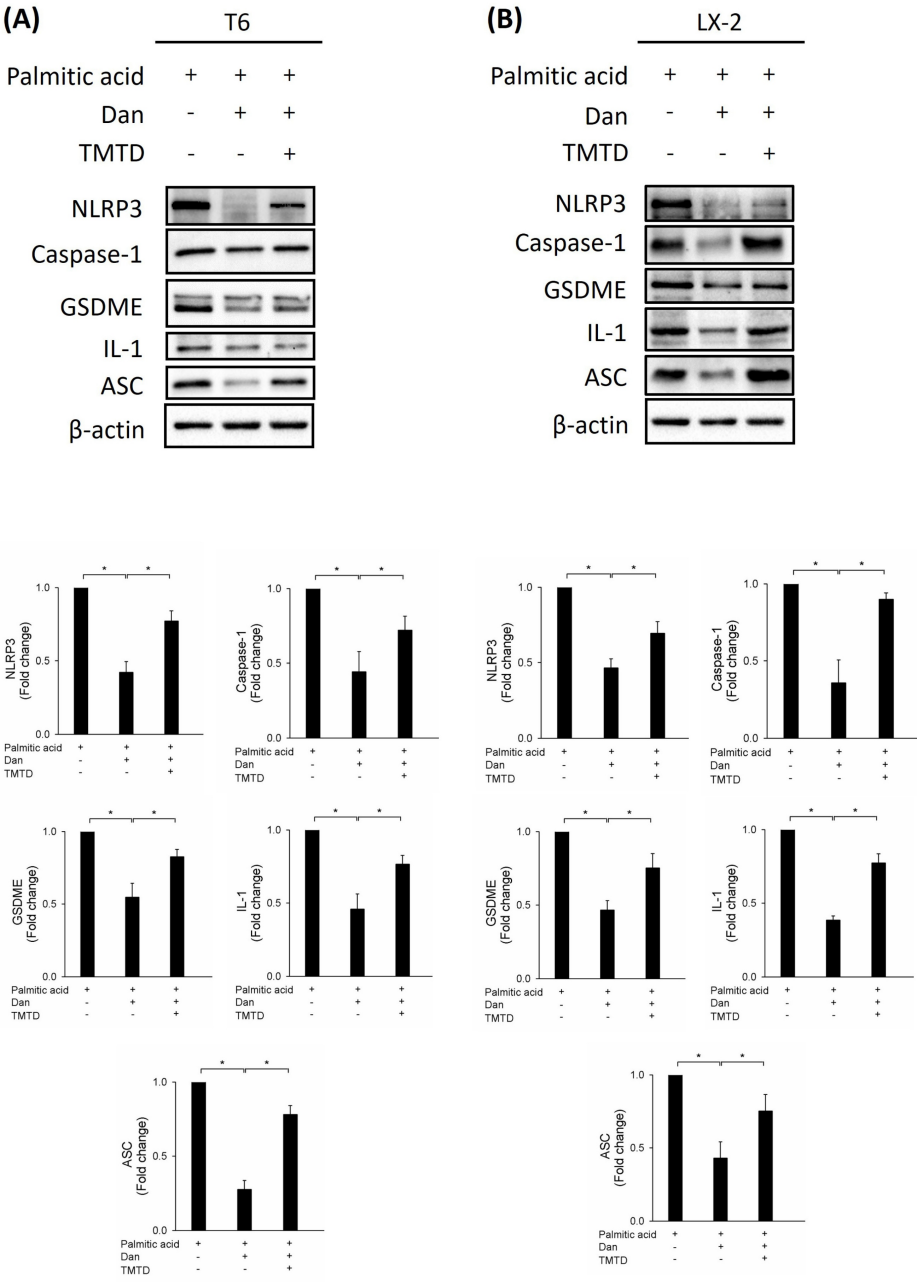
** Effect of tetramethylthiuram disulfide on Danshensu alleviated pyroptosis in palmitic acid (PA)-activated hepatic stellate cells.** Changes in the expression of NLRP3, caspase-1, GSDME, interleukin-1, and ASC in the absence (PA-) or presence (PA+) of 200 μM PA treatments with 30 μM (Dan+) or without (Dan-) Danshensu treatment in (A) T6 and (B) LX-2 cells. Lower panels indicated the quantitative results for specific proteins, which were determined using ImageJ. The data are presented as the mean ± SD from three independent experiments, with significance levels indicated as follows: *P < 0.05.

To study whether Danshensu alleviates pyroptosis in PA-activated T6 and LX-2 cells, we assessed the expression of NLRP3, caspase-1, GSDME, interleukin-1, and ASC by Western blotting. Results showed that the increase in NLRP3, caspase-1, GSDME, interleukin-1, and ASC in PA-activated T6 and LX-2 cells was reversed after treatment with Danshensu. Taken together, these findings suggest that Danshensu has an anti-pyroptotic effect on PA-activated T6 and LX-2 cells (Fig. 3). To further confirm whether Danshensu triggered activated HSCs caused by PA in a pyroptosis-dependent manner, we detected whether the expressions of α-SMA, integrin-β1, and CTGF in PA-activated T6 and LX-2 cells were reversed with Danshensu treatment in the absence or presence of a pyroptosis inhibitor, TMTD (Fig. 4). As shown in Figure 4, the decrease in α-SMA, integrin-β1, and CTGF expressions in PA-activated T6 and LX-2 cells was reversed with Danshensu treatment in the presence of 2 μg/mL TMTD. TMTD also reversed the downregulation of NLRP3, caspase-1, GSDME, interleukin-1, and ASC caused by Danshensu in PA-activated T6 and LX-2 cells (Fig. 5). Thus, Danshensu has an anti-fibrotic role in PA-activated HSCs via pyroptosis.

## Discussion

In this study, we investigated the effects of Danshensu on HSCs activated by PA, focusing on the mechanisms of ROS overproduction and pyroptosis. Our findings indicate that Danshensu plays a significant anti-fibrotic role in PA-activated HSCs, primarily by mitigating ROS production and inhibiting pyroptosis. In a previous study, Danshensu was combined with probiotics, such as *Lactobacillus casei* and *Lactobacillus acidophilus*, to provide anti-methylation and antioxidant properties in a nonalcoholic fatty liver disease animal model 20. Additionally, another study indicated that Danshensu protects against nonalcoholic fatty liver disease by significantly enhanced fatty acid β-oxidation and decreased lipid droplet accumulation in the histone deacetylase 1/TATA-box binding protein associated factor 9-dependent pathway 21. Wang, Su, Xu, and Ko 22 demonstrated that Danshensu attenuated the activation of LX-2 and T6 cells by lipopolysaccharide through modulation of ferroptosis. Moreover, research on Danshensu, a novel inhibitor of indoleamine 2,3-dioxygenase 1, has revealed that it inhibits JAK2-STAT3 signaling, resulting in reduced hepatic fibrosis 23.

ROS can trigger the activation of HSCs, resulting in the accumulation of extracellular matrix proteins. This process contributes to fibrosis, which can progress to cirrhosis and eventually hepatocellular carcinoma 24. In our study, PA treatment led to a marked increase in ROS levels in both T6 and LX-2 cells, suggesting triggered inflammation in PA-activated HSCs. However, Danshensu treatment effectively reduced ROS levels in these cells, indicating its potential to alleviate inflammation in PA-activated HSCs.

Pyroptosis, a form of programmed cell death characterized by inflammasome activation, plays a crucial role in the pathogenesis of liver diseases, including fibrosis 25,26. In our experiments, PA significantly increased the expression of pyroptosis-related proteins NLRP3, caspase-1, GSDME, interleukin-1, and ASC in T6 and LX-2 cells. Interestingly, Danshensu treatment reversed these effects, reducing the expression of these proteins and suggesting an anti-pyroptotic effect. This finding indicates that Danshensu can inhibit pyroptosis in PA-activated HSCs, thereby potentially reducing liver fibrosis.

The activation of HSCs is a key event in liver fibrosis, marked by increased levels of α-SMA, integrin-β1, and CTGF 5,27,28. Our results showed that PA significantly upregulated these markers, confirming HSC activation. Danshensu treatment, however, attenuated the PA-induced upregulation of these fibrotic markers. This suggests that Danshensu not only mitigates ROS production and pyroptosis but also directly inhibits the fibrotic response in HSCs. The findings from this study provide insights into the dual role of Danshensu in combating liver fibrosis. By reducing ROS production and inhibiting pyroptosis, Danshensu addresses two critical pathways involved in the activation and perpetuation of HSCs. This dual action makes Danshensu a promising therapeutic agent for managing liver fibrosis and potentially other related chronic liver diseases. However, *in vivo* studies are needed to confirm the therapeutic potential of Danshensu for liver fibrosis and assess its efficacy and safety in clinical settings. Understanding the precise molecular interactions and identifying potential side effects will be crucial for advancing Danshensu as a treatment for liver fibrosis.

Our study does not address potential side effects or adverse reactions associated with Danshensu treatment. These limitations highlight the need for further research to fully understand the therapeutic potential and safety of Danshensu for treating liver fibrosis in the future. Further research should focus on detailed mechanistic studies to fully elucidate the pathways through which Danshensu exerts its effects on HSCs.

## Conclusions

Our study demonstrates that Danshensu effectively attenuates PA-induced HSC activation by reducing ROS production and inhibiting pyroptosis, thereby offering a potential therapeutic strategy for liver fibrosis. Further investigations are warranted to explore its clinical applications and to fully understand its mechanisms of action.
